# First Record of *Dundubia annandalei* Boulard from Bangladesh, with Predictive MaxEnt Modelling of Climate Change-Driven Range Dynamics (Insecta: Hemiptera: Cicadidae)

**DOI:** 10.3390/insects17080788

**Published:** 2026-07-29

**Authors:** Babu Saddam, Shahamat Jawad, Mohammed Abul Monjur Khan, Cong Wei

**Affiliations:** 1 College of Plant Protection, Northwest A&F University, Yangling 712100, China; bsmalik.malik@gmail.com; 2 Department of Entomology, Faculty of Agriculture, Bangladesh Agricultural University, Mymensingh 2202, Bangladesh; shahamatjawad652@gmail.com (S.J.); khan@bau.edu.bd (M.A.M.K.)

**Keywords:** Hemiptera, Cicadidae, *Dundubia annandalei*, climate change, precipitation, range dynamics, Southeast Asia

## Abstract

This study presents the first record of *Dundubia annandalei* Boulard in Bangladesh and assesses its potential range dynamics through MaxEnt modeling under current and future climate scenarios (under the 2041–2026, 2061–2080, and 2081–2100 time periods). A total of 63 specimens, collected from various locations during field surveys in February–March 2025, were identified based on morphological characteristics. The species distribution model exhibited excellent predictive performance (AUC = 0.967). Precipitation of the warmest quarter (64.4%), elevation (10.8%), and precipitation of the driest month (9.1%) were identified as the most influential environmental factors. Under current climatic conditions, the suitable area is predicted to cover 861,481 km^2^, representing 5.02% of the studied region. Future projections suggest range contraction (SSP585, 2041–2060) and expansion under all chosen emissions scenarios (SSP126 and SSP585), but significant expansion under extreme warming (SSP585), especially during 2061–2080 and 2081–2100. The potential distribution may extend to Bhutan, China, Myanmar, Laos, Nepal, Thailand, Vietnam, Malaysia, Indonesia, and the Philippines. Under climate change scenarios, the results highlight the potential for this species to emerge as a major agricultural concern in the future.

## Simple Summary

This study reports the first record of *Dundubia annandalei* from Bangladesh based on field observations and morphological identification. The species was documented from suitable forest habitats to urban areas, extending its known geographic distribution. In addition, MaxEnt models were used to predict the present and future distributions of this species under climate change scenarios. The results suggest that climatic factors strongly influence habitat suitability and may alter the future range of the species. The findings demonstrate that precipitation is the principal environmental limitation on its survival, ecology, and behavior. Climate change may elicit both detrimental and beneficial responses; it might constrain the range of specific species, although it may also produce contrasting effects, transforming a benign bug into a significant pest. Our study indicates that *Dundubia annandalei* is likely to extend its distribution range into the neighboring country of Bangladesh and could emerge as a potential agricultural and forestry pest due to its polyphagous characteristics in projected climatic scenarios, particularly under SSP585 circumstances. This study provides baseline information for future biodiversity research, management, and conservation planning of cicadas in South Asia and other neighboring countries.

## 1. Introduction

Cicadas serve as valuable subjects for research on speciation and biogeography, as their songs facilitate rapid assessments of local cicada populations and aid in identifying cryptic taxa during the early phases of divergence [1,2]. Cicadas have emerged as an important focus of research regarding endothermy [3,4] and endosymbiosis, as well as remarkable instances of genome evolution [5,6,7]. Additionally, their nanostructured wings have inspired advancements in technology, serving as a model for nanolithography to produce surfaces with hydrophobic, antireflective, and antibiotic properties [8,9,10,11]. Cicadas also act as bioindicators for increasing temperatures [12].

Usually, cicadas are regarded as polyphagous, consuming sap from various host plants during the nymphal and adult stages [13,14,15,16]. During their immature nymph stage, these insects rely on xylem sap and can feed on various hosts, including fruit trees, ornamental plants, and nursery plants [17,18,19]. They can also shift from one host to another when roots intercross in the forest [20,21,22]. Despite this broad host range, these insects are largely ignored by researchers in the study of economic impacts on their host plants. However, numerous studies have demonstrated that several cicada species inflict damage on various crops. For instance, *Guyalna cuta* (Walker, 1850) damages *Jatropha curcas* (commonly known as Barbados nut) in Mato Grosso do Sul Province, Brazil [20]. Similarly, *Quesada gigas* (Olivier, 1790) damages *Schizolobium parahyba* var. *amazonicum* (Ducke) (commonly known as Umbela or Parica) in the Maranhao and Para provinces, Brazil [20,22,23].

In light of climate change, the structural and functional integrity of ecosystems has been negatively impacted in numerous ways [24,25]. Despite significant focus on the impacts of climate change on vertebrates and plants, the effects on the distribution of insects, the most diverse group of animals on our planet, remain a crucial yet largely overlooked area of study [25,26,27]. Recent studies have shown that insects respond acutely to changes in their environment, with shifts in their habitats illustrating both the beneficial and detrimental effects of climate variations [25,28,29]. Notably, the geographic distribution patterns of insects are experiencing considerable changes: they move towards higher latitudes and altitudes, while the population densities of heat-adapted species are increasing [25,27].

Comprehending how organisms react to shifting environments is crucial for anticipating the impacts of climate change on biodiversity and the essential services that specific species provide within ecosystems. There is growing evidence indicating that recent climate warming has influenced the timing of biological events, the geographical distribution of species, and their population dynamics, all of which are closely linked to their physiological limitations. Seasonal outbreaks are observed in various insect groups, including mayflies [29], bush crickets [30], winter moths [31,32], and cicadas [12,33].

The impacts of climate change have escalated dramatically, leading to unprecedented extreme weather events in Bangladesh and surrounding South Asian nations during 2024 and early 2025 [12]. The area is facing increased temperatures, prolonged heatwaves, and intensified flooding, all of which can be directly linked to anthropogenic climate change, rendering these occurrences several folds more probable [12]. Cicadas of Bangladesh, like many other insects in the area, have been largely neglected in research and require contemporary taxonomic, systematic, and phylogenetic analyses. Researchers have conducted a few studies on cicadas in the country [34,35,36,37,38]. Price et al. [39] published an annotated provisional catalog, a regional checklist, and a bibliography on cicadas of Bangladesh, also covering Bhutan, India, Myanmar, Nepal, and Sri Lanka. They documented a total of 189 species in India and Bangladesh [39]. The genus *Dundubia* Amyot & Audinet–Serville, 1843, comprises 33 species globally. However, only one species, *Dundubia ensifera* Bloem & Duffels, 1976, has been previously documented from the Silhet Division of Bangladesh [39]. Considering the diverse ecosystems and abundant habitats for cicadas in Bangladesh, we believe that numerous cicada species have yet to be documented.

During the survey conducted in Bangladesh, we recorded another species of *Dundubia*, i.e., *D. annandalei*, which was extensively observed in orchards of *Mangifera indica* L. (mango), *Litchi chinensis* Sonn. (litchi or lychee), *Selenicereus undatus* (Haworth) (dragon fruit), *Psidium guajava* L. (guava), *Ananas comosus* (L.) Merr. (pineapple), *Musa* sp. (banana), *Artocarpus heterophyllus* Lam. (jackfruit), *Citrus limon* (L.) Osbeck (lemon), and *Citrus sinensis* (L.) Osbeck (orange). Many farmers reported that this cicada (local name: Zhi Zhi Poka) poses a significant threat to these fruits. According to them, the abundance of adults attracts numerous birds, while the nymphs inhibit plant and fruit growth by feeding on xylem sap. In conversations with numerous banana, orange, lemon, and pineapple cultivators in the Madhupur and Mymensingh areas, many farmers expressed their frustration with this species. They noted that the species’ loud calls disrupt their communication while working on the farm and cause headaches at night, making it difficult to obtain adequate rest.

In rural areas of central Bangladesh, many individuals choose to sleep outdoors due to the humidity and temperature, with their homes located among trees such as mango, *Shorea robusta* Gaertn. (sal), *Bambusa balcooa* (Roxb.) (bamboo), jackfruit, and banana. In late March, this species emerges in large numbers following the first rain, and the adults produce loud calling songs that disrupt communication and sleep at night. During the surveys, we recorded a notable amount of exuvia and adult specimens of this species from a variety of tree and shrub species, including sal, bamboo, mango, *Tectona grandis* L.F. (teak), guava, jackfruit, and *Lagerstroemia speciosa* (L.) Pers. (jarul), as well as shrubs such as *Chromolaena odorata* (L.) King & H.E. Robins. (jack), and *Rauvolfia* sp. (rauvolfia).

This paper reports a new addition to the cicada fauna of Bangladesh and provides a comprehensive overview of its distribution in some Asian countries. It also presents observations on its habitat preferences and models its range expansion under current and future climate projections using MaxEnt, a species distribution modeling (SDM) approach. This study investigates the range dynamics of this species under the assumption that future climate change may facilitate its range expansion, potentially turning it into a serious pest of fruit and commercial trees in neighboring countries. These findings highlight the urgent need for in-depth research on insect distribution patterns in relation to climate change, especially across various biogeographical regions with similar habitats.

## 2. Materials and Methods

### 2.1. Data Collection

**Figure 1:**
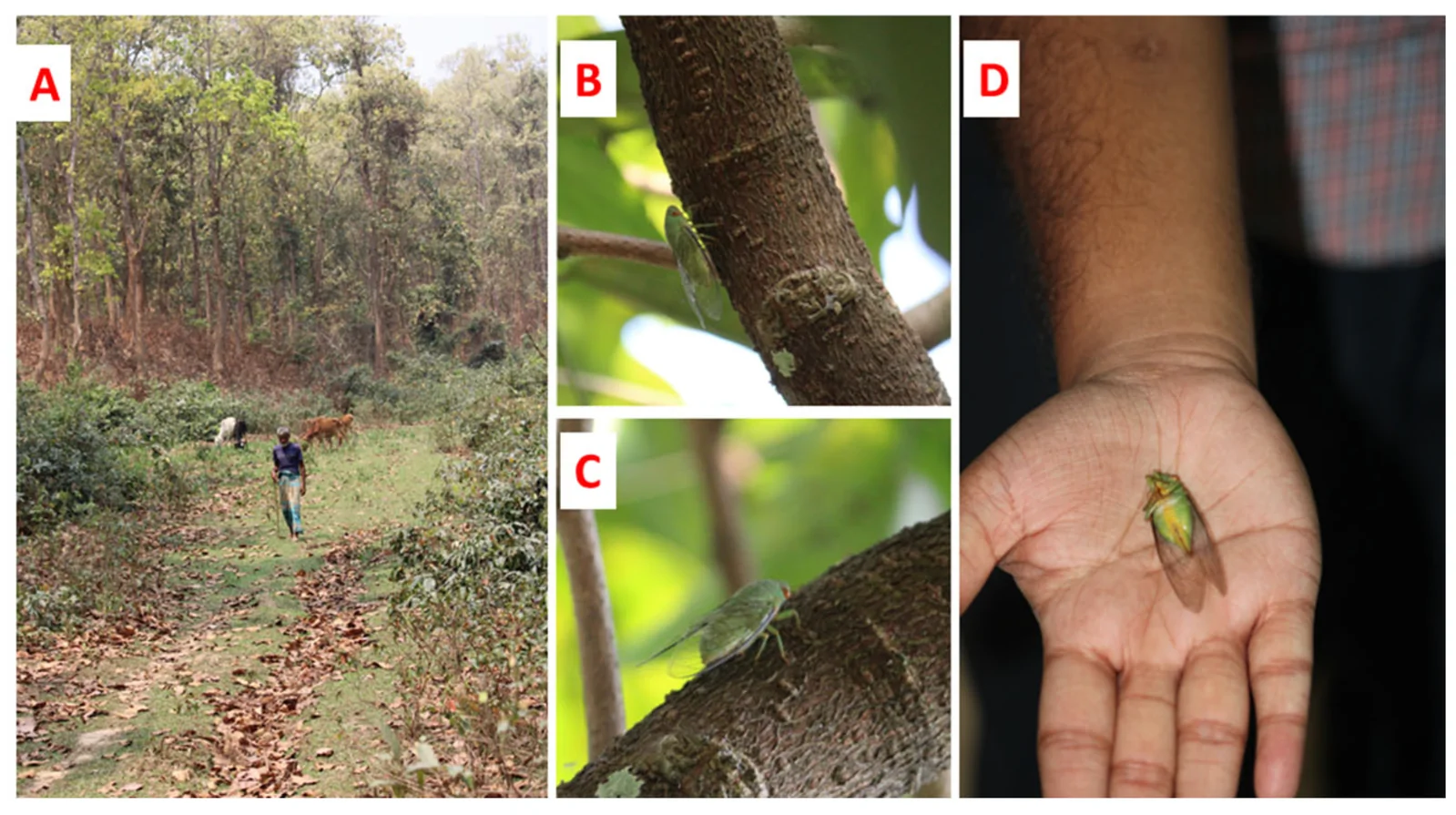
Collection of *Dundubia annandalei*: (**A**) habitat in Madhopur Forest, Bangladesh; (**B**,**C**) lateral view of a live specimen; (**D**) a female collected under a streetlight on the campus of BAU, Mymensingh.

A number of in-depth surveys were carried out in different areas of Bangladesh between February and March of 2025 (Figure 1). Specimens were mostly collected using a sweep net from various localities across the country. A single female specimen was collected under a light pole at Bangladesh Agricultural University (BAU) (Figure 1D). Most cicadas observed in the field were identified through their morphological characteristics. A total of 63 specimens were collected and deposited in the Museum of the Department of Entomology, Bangladesh Agricultural University (BAU), Mymensingh. Individual cicadas were observed and photographed using a Canon EOS–1500D Digital SLR, Tokyo, Japan paired with a Sigma 55–250 mm lens, and their behaviors were meticulously recorded.

**Figure 2:**
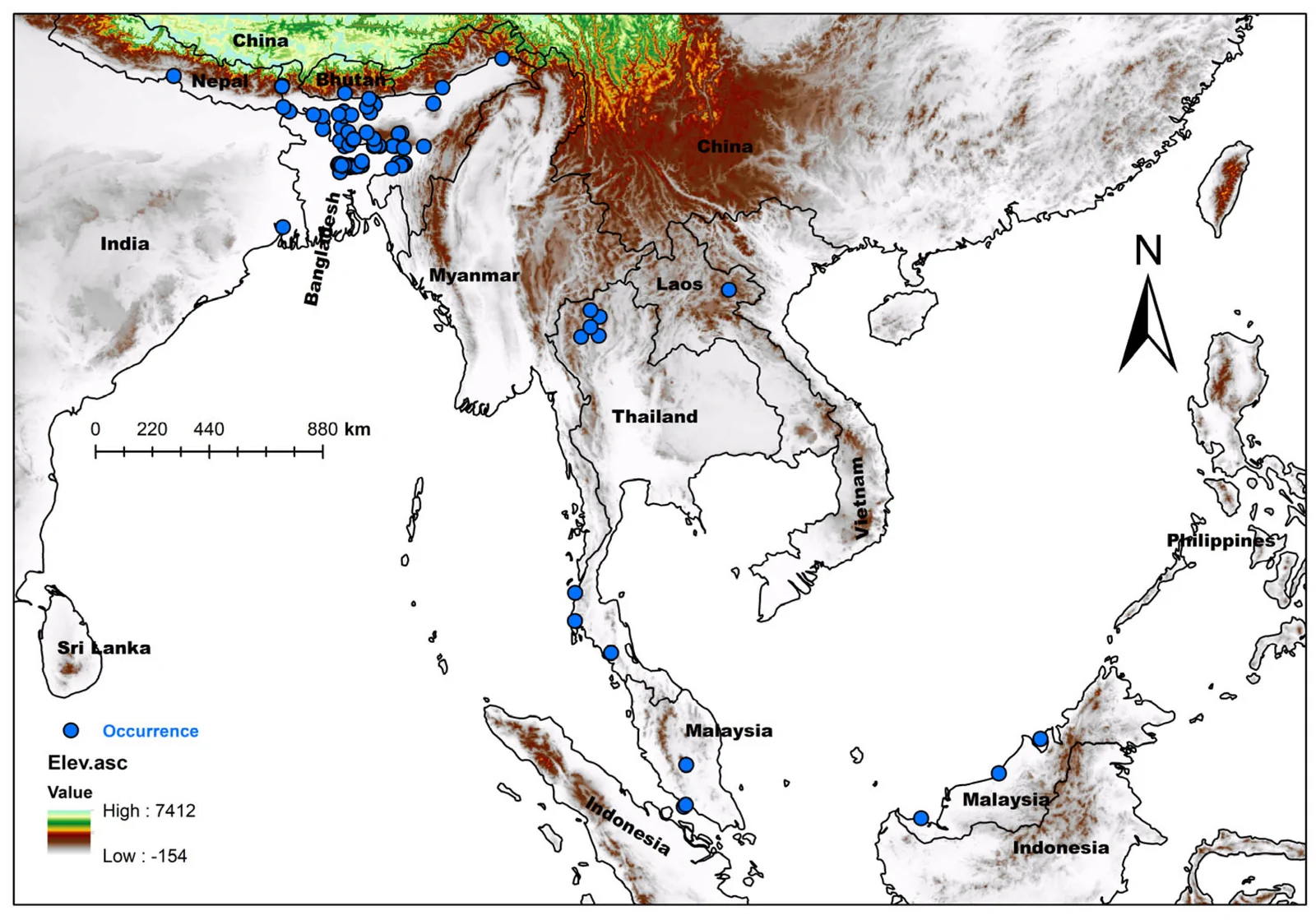
Map showing the geographic locations of *D. annandalei;* ultra pink dots indicating occurrence points after data cleaning and thinning.

All specimens were pinned through the mesonotum, with wings fully extended. Photographs of the habitus were captured using a Canon EF 100 mm macro lens on a Canon 550D (Canon INC, Tokyo, Japan). Images of the identical object at varying focus planes were amalgamated utilizing Helicon Focus 8.2.2 stacking software. Graphic plates were prepared using Adobe Photoshop. The measurements of body parts of adult cicadas were obtained by using specialized tools like digital calipers and image analysis software (ImageJ1) (64-bit Java 1.6.0) software. The description is intended as a supplementary morphological account of the Bangladesh specimen rather than a formal taxonomic revision or redescription of the species. The morphological description followed the terminology of Moulds [40,41]. Locality data were collected using a Garmin GPS MAP 64SC (Olathe, KS, USA) device to document species locations during field investigations. A total of 139 distribution locations were gathered from (i) museum specimens, (ii) surveys, (iii) the Global Biodiversity Information Facility, https://www.gbif.org/occurrence/search?taxonKey=382L9&country=TH, accessed on 1 July 2026, and (iv) published literature [37,39,42] relevant to the same timeframe. Although a significant effort was undertaken to gather occurrence records from surveys, museum-tagged specimens, published literature, and online biodiversity databases, not all documented locales of *Dundubia annandalei* could be included in the study. Certain distribution localities devoid (https://www.gbif.org/occurrence/5858521521, accessed on 1 July 2026) of verified geographic coordinates or inadequate location details for precise georeferencing were omitted to uphold data integrity and diminish geographical ambiguity. Consequently, the current given locations embody the most reliable confirmed occurrence dataset and should be understood with an awareness of this constraint. All these known locations were converted into geocoordinates using Google Earth Pro. To reduce sampling error, a solitary occurrence record was retained in each grid cell to correspond with the spatial resolution of the environmental variables. For modeling objectives, a total of 104 occurrence records for this species were utilized following filtering. Maps were generated using ArcGIS 10.8.2 (Figure 2).

To standardize the model for current climatic conditions, the bioclimatic variables used in this study have a resolution of 2.5 arc-minutes, making them suitable as environmental layers for insect distribution modeling [12]. The Worldclim Global Climate Database (version 2.1; http://www.worldclim.org., accessed on 5 December 2026) was used to obtain 19 bioclimatic variables and one topographic variable for the current environmental layers (1970–2000) (Table 1) and for three future periods (2041–2060, 2061–2080, and 2081–2100) [12,43]. Based on projected future concentrations of greenhouse gases and aerosols, the IPCC (International Panel on Climate Change) designed multiple potential scenarios, known as shared socio-economic pathways (SSPs): SSP126 (green), SSP245 (intermediate), SSP370 (high), and SSP585 (very high) [44,45]. For the first time, we considered two of these scenarios: SSP126 (green) and SSP585 (very high) [46]. Data processing and generating maps were performed using ArcGIS software (Ver. 10.8.2; http://www.arcgis.com).

### 2.2. Selection of Study Area

*Dundubia annadalei* has previously been documented in India, Thailand, and Malaysia [39]. We selected these neighboring countries to assess the impact of climate change on the potential distribution of this species for two reasons: (i) Bangladesh shares similar climatic zones, vegetation types, and agro-ecosystems with these countries, making the transboundary spread of insect species ecologically feasible; (ii) this species may become a potential agricultural and forestry pest in neighboring countries, especially under future climate scenarios, necessitating a regional-scale risk assessment.

### 2.3. Environmental Variables Selection and MaxEnt Model Settings

Pearson correlation coefficients for the 20 variables (19 bioclimatic variables and elevation) for the current period (1970–2000) were calculated using the Ecological Niche Modeling (ENM) tool to reduce bias caused by multicollinearity (Table 1). When the absolute value of Pearson’s correlation between two variables exceeded 0.85, one of the two variables was eliminated [12,45,47]. After this selection process, followed by Pearson’s correlation, eight environmental variables (Bio2, Bio3, Bio4, Bio9, Bio12, Bio14, Bio15, and Bio18) and one topographic variable (elevation) were retained for use in the modeling (Table 1). Because it performs precisely when the sample size (location number) is small, the subsample method was chosen for model calibration.

For the present study, initially three GCMs (Global Climate Models), BCC–ESM2–MR, MIROC6 (Model for Interdisciplinary Research on Climate, version 6), and CMCC–ESM2 (Centro Euro–Mediterraneo sui Cambiamenti Climatici–Earth System Model version 2), were tested. Based on the results’ performance, only one model, BCC–ESM2–MR (Beijing Climate Centre, China Meteorological Administration), was selected. The last two models failed to provide suitable results, which were unfavorable for the designed study area. The BCC–ESM2–MR (Beijing Climate Centre, China Meteorological Administration) model performs well in simulating temperature and precipitation patterns across monsoon-dominated regions, making it suitable for Bangladesh and adjacent countries, and is widely adopted in biodiversity and pest risk modeling, ensuring methodological consistency and comparability with previous climate change impact studies. The MaxEnt model was configured following Saddam and Wei [12].

### 2.4. Classification of Suitable Habitats and Distribution Range Estimation

We employed ArcGIS and the SDM Toolbox to illustrate range changes for *D. annandalei* under prospective climatic scenarios. The ETAS (equal training sensitivity and specificity) threshold classified the distribution results into four categories: unsuitable distribution ranges (0–0.35), lowly suitable distribution ranges (0.35–0.62), moderately suitable distribution ranges (0.62–0.79), and highly suitable distribution ranges (0.79–1). The Zonal Statistics tool in ArcGIS 10.8.2 was employed to assess the potential ranges and suitability classifications for the species across several climate scenarios. Subsequently, we analyzed the current suitable distribution against the suitable distribution projected under certain future climate scenarios to determine changes in distribution ranges (contraction and expansion). Changes between present and future forecasts were examined utilizing the distribution changes between the binary SDMs tool within the SDM toolbox [48].

## 3. Results

### 3.1. Dundubia annandalei Boulard, 2007

*Measurements* (in mm; 10♂): Forewing: 37.98–38.16; hindwing: 20.84–20.93; width of the head: 10.14–10.17; width of the pronotum: 11–11.1; length of the pronotum: 4.30–4.32; width of the mesonotum: 9.25–9.28; length of the mesonotum: 5.43–5.50; length of the metanotum: 0.9–1.02; length of the abdomen: 14.48–14.58; length of the proboscis (length of the rostrum, including the labrum and mentum): 5.19–5.24.

*Description of Male* (Figure 3A–D)
  Head (Figure 3A): Head entirely leaf green with orange to golden eyes and pale sanguine ocelli; anteclypeus and mentum, leaf green; labium of the rostrum, brown, turning darker towards the piercing end. The pedicel of the antenna typically exhibits a green hue, complemented by a brownish second flagellomere. However, in individuals that have recently emerged, the entire antenna takes on a green appearance as it matures in color. Head slightly narrower than mesonotum. Vertex ochraceous, with darker infuscation around the ocellar region. Compound eyes are prominent. Ocelli arranged in a triangular configuration; median ocellus situated anterior to lateral ocelli. Postclypeus moderately swollen, transversely grooved; ochraceous with darker lateral fasciae. Anteclypeus smaller, concolorous with postclypeus. Rostrum extending posteriorly to about the middle coxae.
**Figure 3:**
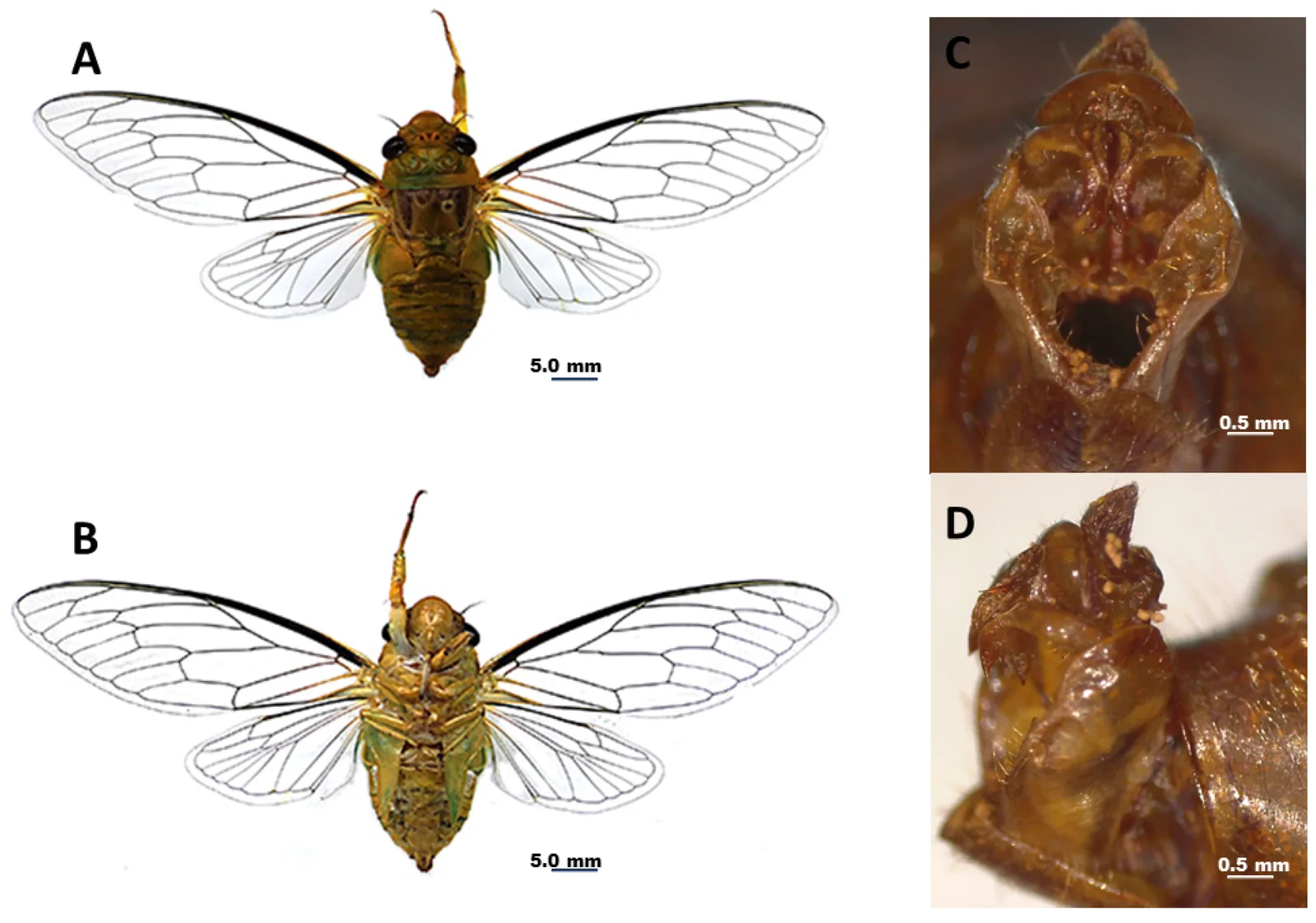
*Dundubia annandalei* Boulard, 2007, male: (**A**) habitus—dorsal view; (**B**) ventral view; (**C**) male pygofer, ventral view; (**D**) male pygofer, lateral view.

 

*Thorax* (Figure 3A): The thorax exhibits a vibrant green hue, reminiscent of foliage. The recently emerged adult’s specimens exhibit tiny silvery hairs across their bodies, giving them a grayish–green appearance, which eventually transitions to a leaf green adorned with yellow patches as they approach the completion of their lifecycle. The body color of the dried pinned specimens transitions to an ochraceous shade after a span of 3–4 days. Pronotum slightly narrower than mesonotum; lateral pronotal collar distinct and moderately expanded. Pronotal collar ochraceous-brown with darker median and paramedian markings. Mesonotum wider than pronotum, bearing characteristic dark fasciae surrounding cruciform elevation. Cruciform elevation distinct, elevated, posteriorly produced. Metanotum is concealed dorsally.

 

*Legs* (Figure 3A,B): The base of the legs, coxa, and trochanter are always yellow, irrespective of age, and so is the tip of the leg; tarsus and claw are yellowish-brown. Fore femora are robust, with distinct primary and secondary spines adapted for grasping. Tibiae are slender, ochraceous-brown. Tarsi are three-segmented with darkened pretarsal claws.
  *Wings* (Figure 3A,B): Hyaline. Completely transparent. The basal veins are body color, which turns darker towards the marginal area. In older specimens, the basal vein of the forewing can turn greenish-yellow or completely yellow. The radius and subcostal vein are dark roseate brown to black. Fore wings hyaline, elongate, with well-developed venation; costal margin is slightly infuscate. Basal cell, pale ochraceous. Radial and radiomedial crossveins are weakly marked.

 

*Abdomen* (Figure 3B): The abdomen is mostly the same green color as the rest of the body, except the median part of all eight sternites, which varies from yellowish-green to rich yellow. Abdomen robust, longer than broad, tapering posteriorly. Dorsal tergites dark ochraceous to brown with segmental banding. Timbal covers present laterally at basal abdominal segments. Ventral abdomen paler, with visible sternites and faint median markings. Sternites III to VI translucent. Terminal abdominal segment rounded posteriorly. The opercula are elongated, comma-shaped, with a prominent ridge.

 

*Male genitalia* (Figure 3C,D): Upper lobe of pygofer reduced, exposing the base of the clasper in adults. The clasper has two pointed lobes split vertically, making it appear as an inverted ‘Y’ from the side, giving an impression of the claw of a crab. Each of these lobes is further bifurcated into two outwardly curved pointed projections, forming a structure of an inverted ‘V’ towards the distal end. Median lobe is attached to the upper part of the clasper, leaving an opening for the head of the aedeagus. Anal tube and anal style are prominent.

### 3.2. MaxEnt Model Performance and Importance of the Environmental Predictors

The AUC value of the SDM obtained was 0.967 after a model run for *D. annandalei*, indicating that the model’s performance exceeded 8 and was classified as excellent (Figures S1 and S2). This demonstrates robust model efficacy and implies that the predicted results are in close alignment with real-world data. The significance of the predictors for the baseline distribution models varies according to the species involved and its presence in the particular region, including the habitat involved. For instance, the main variable in the species distribution model of *D. annandalei* was the precipitation of the warmest quarter (Bio 18), followed by elevation, precipitation of the driest month (Bio 14), isothermality (BIO2/BIO7) (_100) (Bio 3), precipitation seasonality (coefficient of variation) (Bio 15), temperature seasonality (standard deviation _100) (Bio 4), mean temperature of the driest quarter (Bio 9), annual precipitation (Bio 12), and the mean diurnal range (mean of monthly [max temp _min temp]) (Bio 2). In the analysis, the four primary environmental variables that significantly influenced the performance of the MaxEnt model were the precipitation of the warmest quarter (64.4%), elevation (10.8%), precipitation of the driest month (9.1%), and isothermality (BIO2/BIO7) (_100) (7.7%) (Table 1). The combination of these four environmental variables contributed a significant 92% to the overall development of the model.

### 3.3. Predicted Habitat Suitability and Potential Ranges Under the Current Climate Scenarios

**Figure 4:**
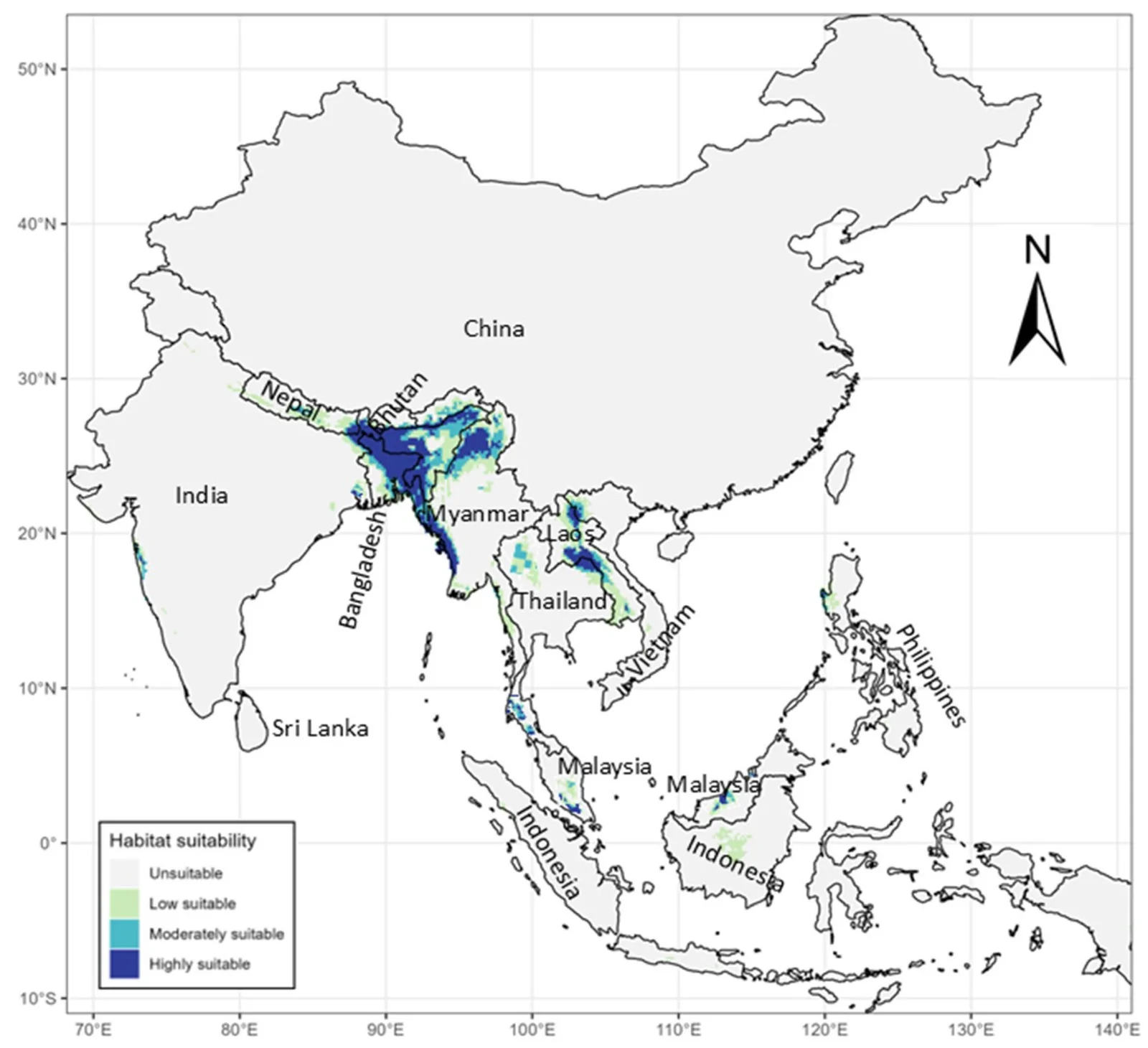
Potential suitable areas predicted by the MaxEnt model under the current climate scenario. The highly suitable habitats were majorly distributed in Bangladesh, Bhutan, India, Laos, Myanmar, Nepal, Thailand, and Vietnam and, minorly distributed in Malaysia and the Philippines.

**Figure 5:**
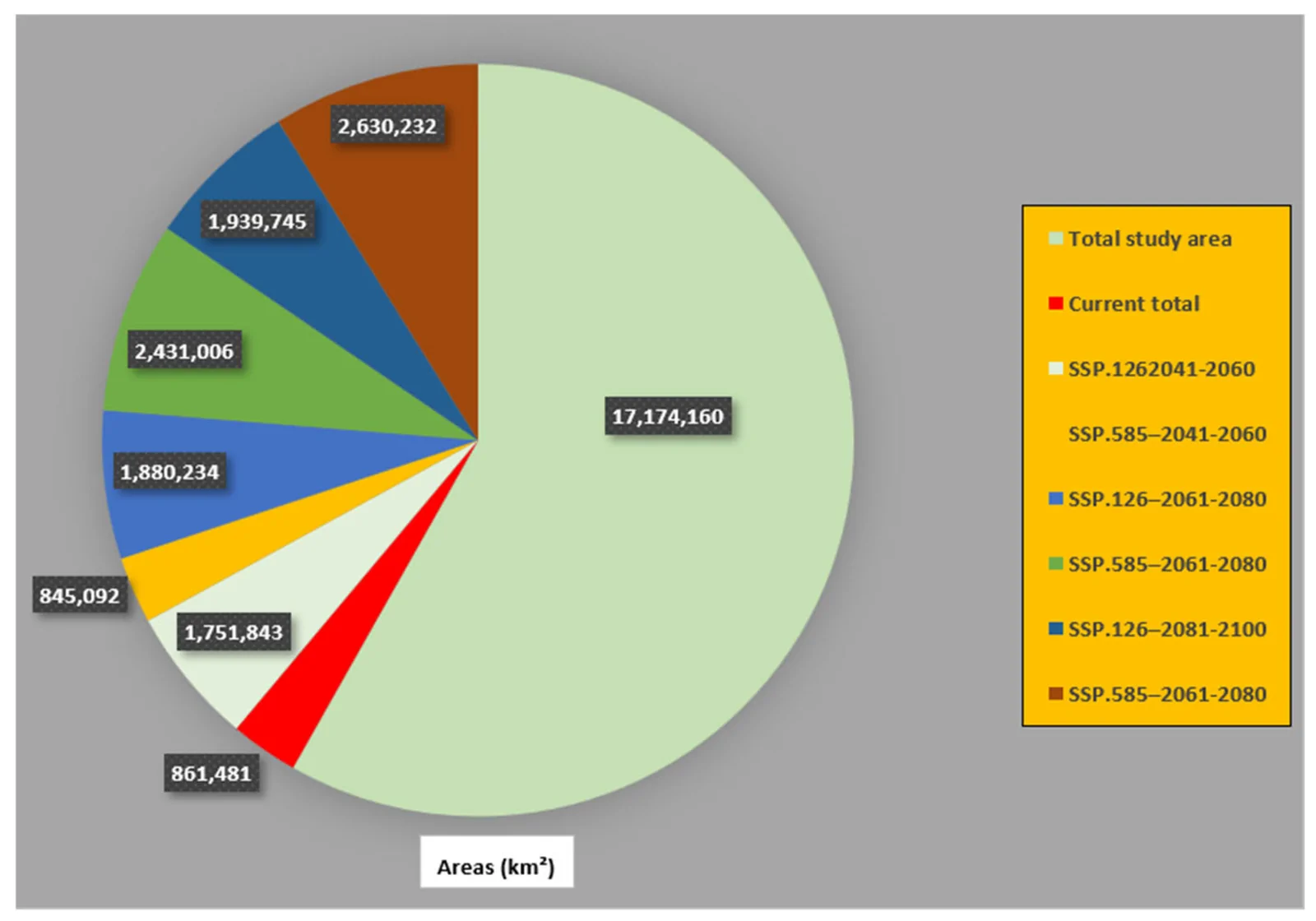
Total study area and suitable areas under different climate scenarios.

Prior studies confirm that this species has been documented in Malaysia, Thailand, and India. This species has been documented for the first time in Bangladesh. According to current climatic projections, *D. annandalei* is expected to have a predicted habitat suitability in neighboring countries, such as Bhutan, Indonesia, Myanmar, Nepal, the Philippines, Vietnam, and Laos, based on the ETSAS threshold under current climate conditions (Figure 4). It is interesting to observe that this species favored habitats with higher precipitation levels and ranges that correspond to coastal areas. The total potential distribution range of *D. annandalei* was projected to be 861,481 km^2^, which was estimated to occupy 5.02% of the study area under the current climate scenario. The highly suitable habitats were projected to be 298,928 km^2^, which was estimated to occupy 1.74% of the total study area (Figure 4 and Figure 5). The moderately suitable habitats were projected to be 178,957 km^2^ (Figure 4 and Figure 5), which were estimated to occupy 1.05% of the total study area, respectively (Figure 4). The highly suitable and moderately suitable habitats were mainly distributed in Bangladesh, Bhutan, India, Laos, Myanmar, Malaysia, Nepal, Philippines, Thailand, and Vietnam (Figure 4).

### 3.4. Predicted Habitat Suitability and Potential Ranges Under Future Climate Scenarios

**Figure 6:**
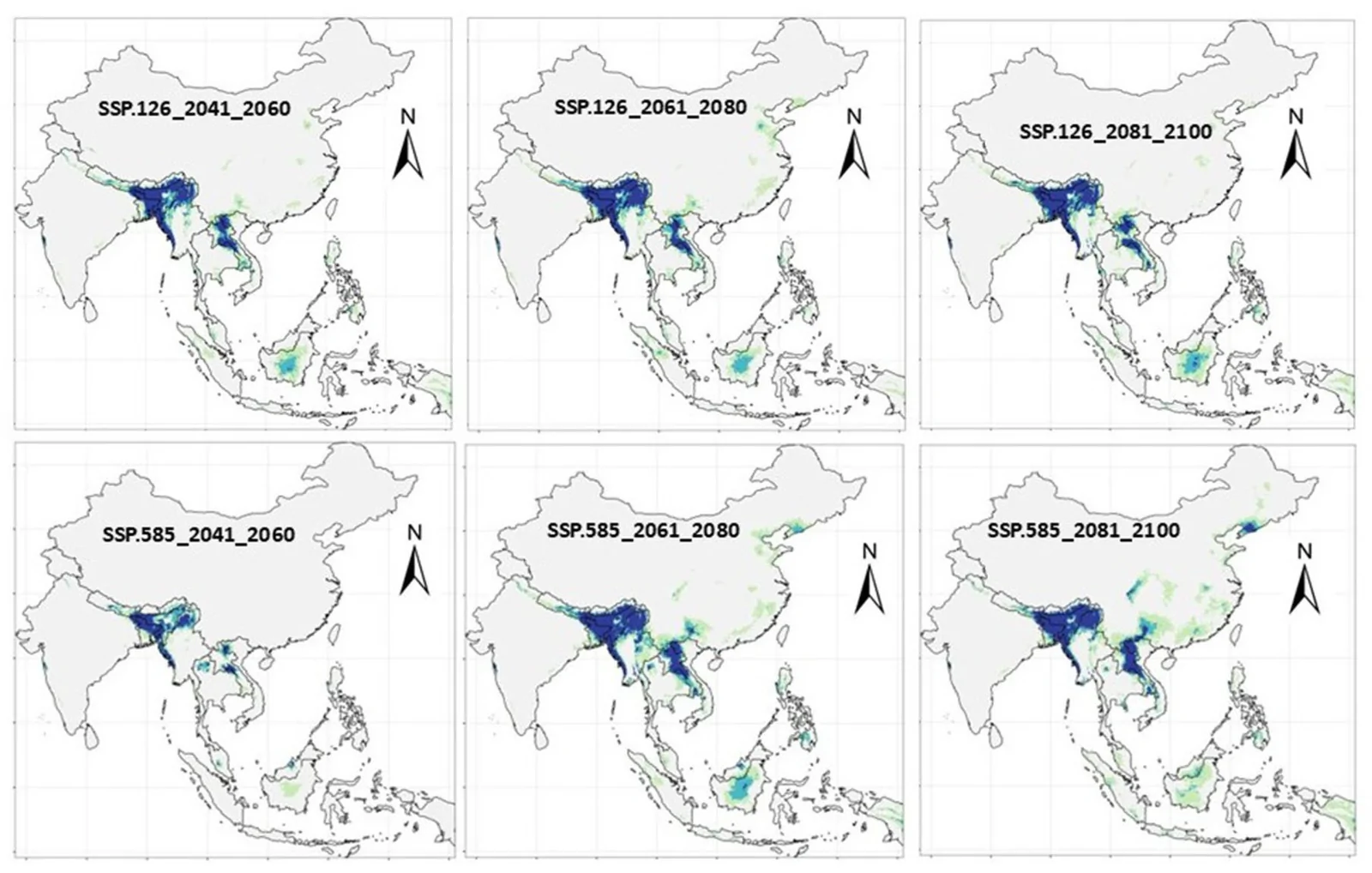
Potential suitable areas predicted under different future climate scenarios by the MaxEnt model.

The predicted suitable distribution ranges of *D. annandalei* exhibit considerable variability across several SSPs in projected climate scenarios (Figure 5 and Figure 6) compared to the current climate scenario. Across all climate scenarios (2041–2060, SSP126; 2061–2080, SSP126; 2061–2080, SSP585; 2081–2100, SSP126; and 2081–2100, SSP585), except for 2041–2060, SSP585 (Figure 5 and Figure 6), there was a notable increase in the distribution ranges of *D. annandalei* within the designated study region. However, according to the 2041–2060 SSP585 climate scenario, there is a noteworthy decrease, around 16,389 km^2^, in the distribution range of *D. annandalei* in the designated study region (Figure 5 and Figure 6). The total potential suitable habitat of *D. annandalei* was projected to be 1,751,843 km^2^, estimated to occupy 10.21% of the study area for the period 2041–2060, SSP126. The total potential suitable habitat of *D. annandalei* was projected to be 845,092 km^2^, 1,880,243 km^2^, 2,431,006 km^2^, 1,939,745 km^2^, and 2,630,232 km^2^, which was estimated to occupy 4.93%, 10.95%, 14.16%, %, 11.3%, and 15.32% in the study area under the 2041–2060, SSP585; 2061–2080, SSP126; 2061–2080, SSP585; 2081–2100, SSP126; and 2081–2100, SSP585, respectively (Figure 6 and Table 2). The habitat suitability under these time frames and climate scenarios will result in the species being found in countries like Bangladesh, Bhutan, China, India, Indonesia, Laos, Myanmar, Malaysia, Nepal, the Philippines, Thailand, and Vietnam. This is due to the fact that temperature and higher precipitation will affect the distribution ranges, thereby confining this species within certain boundaries (Figure 6). The boundaries of Bangladesh, Bhutan, India, Laos, Nepal, Thailand, and Vietnam contain the largest portion of highly suitable habitats.

### 3.5. Predicted Range Dynamics Under Future Climate Scenarios

The range expansions of *D. annandalei* under selected scenarios were restricted to the study area. However, range expansions under the 2061–2080 (SSP585) and 2081–2100 (SSP585) scenarios overlapped significantly in Bangladesh, Bhutan, China, Laos, Myanmar, Nepal, Thailand, Vietnam, a small part of India, a small portion of Malaysia, and some islands of Indonesia and the Philippines (Figure 6). However, under the 2081–2100 (SSP585) scenario, a significant range expansion is noted, particularly in China (Figure 6). Range contraction under the 2041–2060 (SSP585) scenario occurred in scattered regions in Bangladesh, Bhutan, India, Indonesia, Laos, Myanmar, Malaysia, Nepal, Thailand, and Vietnam (Figure 6). The stable ranges under all scenarios overlapped in Bangladesh, Bhutan, India, Laos, Myanmar, Malaysia, Vietnam, and Thailand (Figure 6).

Under the 2041–2060; 2061–2080, and 2081–2100 climate scenarios, the suitable distribution ranges were predicted to be increased by 890,362 km^2^ (SSP126. 2041–2060), 1,018,753 km^2^ (SSP126. 2061–2080), 1,569,525 km^2^ (SSP585. 2061–2080), 1,078,264 km^2^ (SSP126. 2081–2100), and 1,768,751 km^2^ (SSP585. 2081–2100) in the study area, respectively. However, according to the 2041–2060 SSP585 climate scenario, there is a noteworthy decrease, around 16,389 km^2^, in the distribution range of *D. annandalei* in the selected study region (Figure 5 and Figure 6 and Table 2). Based on the projections of these three climate scenarios, *D. annandalei* has the potential to expand its range into major parts of Bhutan, China, Indonesia, Laos, Myanmar, Nepal, the Philippines, Thailand, and Vietnam, as well as the Himalayan regions of India (Figure 6). Under the 2081–2100 climate scenario, the suitable distribution ranges were predicted to increase by 1,768,751 km^2^ (SSP585) in the study area (Table 2), which is more than twice the distribution ranges predicted under the current climate scenario.

## 4. Discussion

*D. annandalei* is distinguished from all other species by its consistent grass-green hue and the comma-shaped male opercula [49,50]. This cicada has perplexed numerous researchers, owing to its physical resemblances with *Dundubia terpsichore* (Walker, 1850). However, native, fresh specimens of *Dundubia annandalei* are distinguished from all other species by their consistent grass-green hue, whereas dry specimens turn brown and may therefore be confused with *D. terpsichore*. The twin species were originally believed to be differentiated solely by male voice; however, upon examining both cicadas in their natural habitat, Boulard [49] discovered that *D. annandalei* appears teneral and uniformly green when alive, turning brown in pinned specimens, in contrast to *D. terpsichore*, which remains brown whether alive or deceased [49,50].

The cicadas found in Bangladesh, particularly those belonging to the genus *Dundubia*, have not received adequate attention and necessitate contemporary taxonomic, systematic, and phylogenetic examination. Previous investigations have recorded cicada species in Bangladesh and adjacent nations, resulting in a comprehensive catalog of 189 species [39,49]. The recently recorded species, *D. annandalei*, displays unique morphological traits such as the clasper, which has two pointed lobes split vertically, making it appear as an inverted ‘Y’ from the side, giving an impression of the claw of a crab; each of these lobes are further bifurcated in two outwardly curved pointed projections, forming a structure of an inverted ‘V’ toward the distal end. The specimens have been collected from multiple locations, including the Mymensingh Division, where thorough surveys were carried out. The range of *D. annandalei* encompasses a variety of habitats, showcasing its adaptability, and it has been thoroughly documented in both urban and natural environments throughout Bangladesh. During the surveys in Bangladesh, we documented a significant quantity of exuvia and adult specimens of *D. annandalei* from several tree species, including sal, bamboo, mango, teak, guava, jackfruit, and jarul, as well as shrubs such as jack and rauvolfia, which may serve as host plants for this species.

Plenty of research indicates that human activity and climate change are key influences on the range dynamics of insects [51,52,53,54]. Our study showed that *D. annandalei* is expected to have a predicted suitable distribution range in neighboring countries, such as Bhutan, China, Indonesia, Myanmar, Nepal, the Philippines, Vietnam, and Laos, based on the ETSAS threshold under the current climate conditions. Anticipated climate changes, particularly increased atmospheric carbon dioxide levels and temperatures, along with fluctuating precipitation patterns, influence the biology and ecology of specific insects, notably invasive species, which can significantly threaten crop production [54]. The environmental factors that notably affected the distribution ranges of *D. annandalei* were precipitation of the warmest quarter, elevation, precipitation of the driest month, and isothermality (BIO2/BIO7). Precipitation will significantly influence the spread of this species in neighboring countries. A significant effort was undertaken to gather occurrence records from surveys, museum-tagged specimens, published literature, and online biodiversity databases; nonetheless, not all documented locales of this species could be included in the study for present and future prediction. Certain distribution localities devoid of verified geographic coordinates or inadequate location details for precise georeferencing were omitted to uphold data integrity and diminish geographical ambiguity. Consequently, the current model embodies the most reliable confirmed occurrence dataset and should be understood with an awareness of this constraint.

The projected total potential distribution range of *D. annandalei* is 861,481 km^2^, which was calculated to occupy 5.02% of the research area under the current climatic conditions. The present climate scenario forecasts highly and moderately favorable distribution ranges in Bangladesh, China, India, Indonesia, Laos, Malaysia, Nepal, Thailand, and Vietnam, including a very few suitable range in China.

Insect species are altering their distribution as a result of climate change, as well as the rise in international trade, which facilitates their spread across the globe. When it comes to agricultural insect pests, such a change in dispersal patterns can significantly impact agricultural output [55,56]. The spatial distribution and population levels of all living organisms are highlighted by the unique climatic needs of each species, which are essential for their growth, development, reproduction, and survival. Altered temperature and precipitation trends, along with anticipated climate shifts, will influence the distribution, survival, and reproduction of species moving forward [57,58,59,60]. The range expansions of *D. annandalei* in the chosen scenarios were confined to the study area. Nonetheless, the range expansions projected for the periods of 2061–2080 and 2081–2100 under the SSP585 scenarios show considerable overlap in regions such as Bangladesh, Bhutan, China, Laos, Myanmar, Malaysia, Nepal, Thailand, Vietnam, India (Western Ghats), as well as certain islands in Indonesia and the Philippines. Range contraction during the periods of 2041–2080 under the SSP585 scenarios was observed in various regions across Bangladesh, Bhutan, Indonesia, Laos, Myanmar, Malaysia, Nepal, Thailand, and Vietnam. The stable ranges across all scenarios were found to coincide in Bangladesh, India, Myanmar, Vietnam, and Thailand.

A limitation of the present study is that few reported places of *D. annandalei* could not be included due to the lack of availability of verified coordinates. Thus, the estimated current and future distributions should be considered preliminary estimations based on the best available verified occurrence data. Additional georeferenced records and recent locality surveys in the neighboring country of Bangladesh or from undersampled locations and future surveys could increase model accuracy and allow further refinement of forecasts of the species’ potential distribution under changing climatic circumstances. Regardless of this constraint, the model provides useful information on the probable climatic suitability and range dynamics of *D. annandalei*, emphasizing areas that may be of interest for future surveys and management.

## 5. Conclusions

This study represents a pioneering effort to explore the range dynamics of this species in the context of current and future climate conditions across South Asia and its neighboring regions. The MaxEnt projections indicate that the future potential distribution of *D. annandalei* may vary depending on the climate scenario and time period considered. Under SSP126, suitable habitat is predicted to remain relatively stable with localized changes. However, under SSP585, substantial shifts in habitat suitability are projected, resulting in regional expansion in some areas and contraction in others. The predicted habitat suitability for this species is expected to rise in more countries than currently documented, indicating potential escalating agricultural threats ahead. This research enhances our comprehension of the impacts that climate and habitat alterations may have on cicada populations and their potential for range expansions.

## Figures and Tables

**Table 1:** In bold, environmental variables were selected for analysis after Pearson’s correlation coefficient and the percent contribution rate of variables in model building were obtained.

Variable	Description	Percent Contribution
**BIO1**	**Annual mean temperature**	
**BIO2**	**Mean diurnal range (mean of monthly [max temp _min temp])**	**0.7**
**BIO3**	**Isothermality (BIO2/BIO7) (_100)**	**7.7**
BIO4	Temperature seasonality (standard deviation _100)	2.3
BIO5	Max temperature of warmest month	
BIO6	Min temperature of coldest month	
BIO7	Temperature annual range (BIO5–BIO6)	
BIO8	Mean temperature of wettest quarter	
BIO9	Mean temperature of driest quarter	1.2
BIO10	Mean temperature of warmest quarter	
BIO11	Mean temperature of coldest quarter	
**BIO12**	**Annual precipitation**	**1.2**
BIO13	Precipitation of wettest month	
**BIO14**	**Precipitation of driest month**	**9.1**
**BIO15**	**Precipitation seasonality (coefficient of variation)**	**2.9**
BIO16	Precipitation of wettest quarter	
BIO17	Precipitation of driest quarter	
**BIO18**	**Precipitation of warmest quarter**	**64.4**
BIO19	Precipitation of coldest quarter	
	**Elevation**	**10.8**

**Table 2:** Total suitable area and range dynamics of *D. annandalei* under different climate scenarios.

Scenarios	Total Suitable Area (Km^2^)	Decreased Area (Km^2^)	Increased Area (Km^2^)
Current	861,481		
SSP126. 2041–2060	1,751,843		890,362
SSP585. 2041–2061	845,092	16,389	
SSP126. 2061–2080	1,880,234		1,018,753
SSP585. 2061–2080	2,431,006		1,569,525
SSP126. 2081–2100	1,939,745		1,078,264
SSP585. 2081–2100	2,630,232		1,768,751

## Data Availability

The data presented in this study are available as Supplementary Material. The raw data supporting the conclusions of this article will be made available by the authors on request.

## Supplementary Materials

The following supporting information can be downloaded at: https://www.mdpi.com/article/10.3390/insects17080788/s1, Figure S1: Receiver operating characteristic (ROC) curves and the area under the curves (AUC) values of the final habitat suitability models of *D. annandalei*; Figure S2: Jackknife results of the final habitat suitability models of *D. annandalei*.
