# Supplementary material for: First Record of Dundubia annandalei Boulard from Bangladesh, with Predictive MaxEnt Modelling of Climate Change-Driven Range Dynamics (Insecta: Hemiptera: Cicadidae)
Source: Insects. 2026 Jul 29;17(8):788. doi: 10.3390/insects17080788 (PMC13513301; doi:10.3390/insects17080788)
Supplement: Supplementary file 1 [file insects-17-00788-s001.zip › insects-4279596-supplementary.pdf]

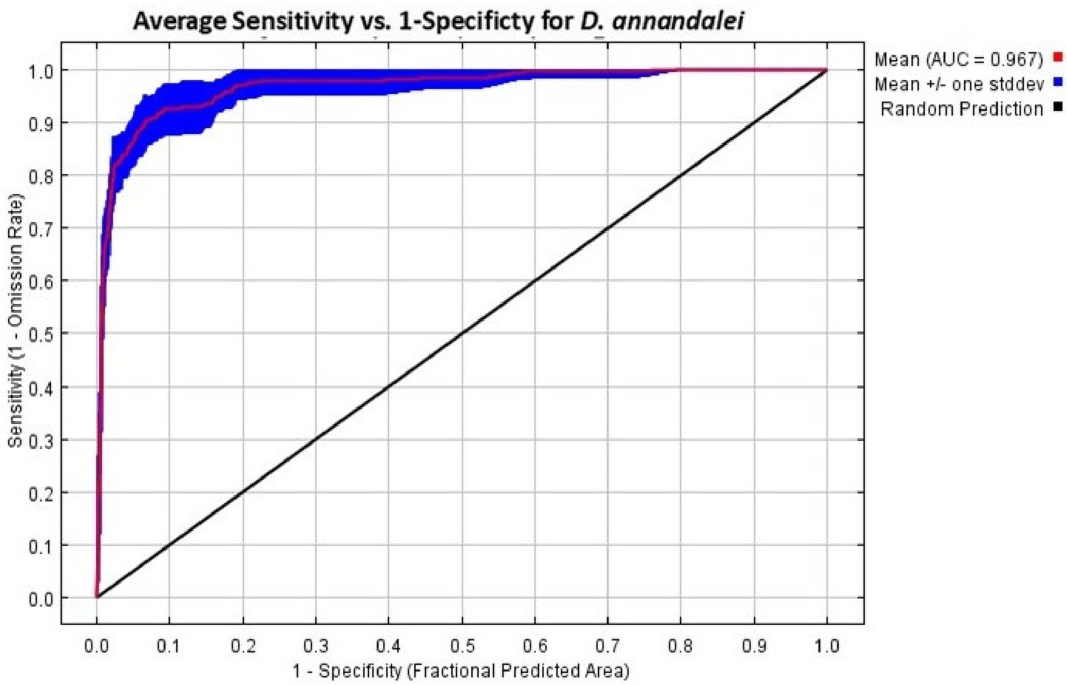


**Figure S1.** Receiver operating characteristic (ROC) curves and the area under the curves (AUC) values of the final habitat suitability models of *D. annandalei*.

Receiver Operating Characteristic (ROC) curve showing the performance of the MaxEnt species distribution model for *Dundubia annandalei*. The x-axis represents 1 − Specificity (fractional predicted area or false positive rate), while the y-axis represents Sensitivity (1 − omission rate or true positive rate). The red line indicates the mean model performance across replicate runs (Mean AUC = 0.967), the blue shaded area represents the mean ± one standard deviation among replicate model runs, and the black diagonal line represents random prediction performance.

The figure demonstrates that the MaxEnt model has very high predictive accuracy, as indicated by the Area Under the Curve (AUC) value of 0.967. The ROC curve lying well above the random prediction line indicates that the model effectively distinguishes suitable from unsuitable habitats for *D. annandalei*. The narrow spread of the blue confidence band further suggests consistency and stability of the model predictions across replicates

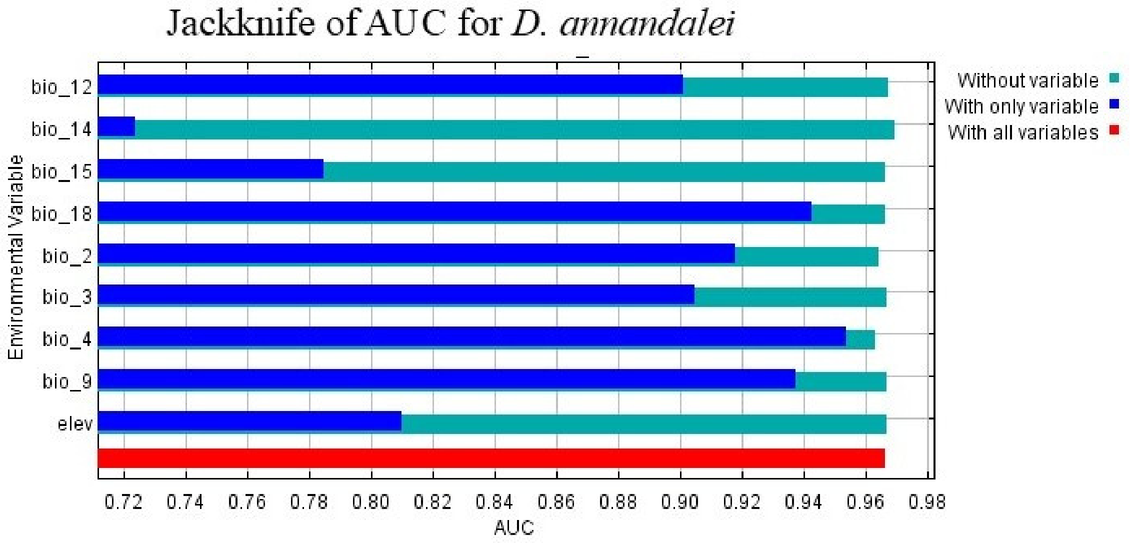


**Figure S2.** Jackknife results of the final habitat suitability models of *D. annandalei*.

Jackknife test of Area Under the Curve (AUC) values showing the relative contribution of environmental variables to the MaxEnt model predicting the distribution of *Dundubia annandalei*. Cyan bars ("Without variable") represent model performance when a variable is omitted; blue bars ("With only variable") represent model performance when the model is run using only that variable; and the red bar ("With all variables") represents the performance of the full model including all variables.
